# A Rare Case of Primary Breast Diffuse Large B-cell Lymphoma in an Acquired Immunodeficiency Syndrome Patient

**DOI:** 10.7759/cureus.36019

**Published:** 2023-03-11

**Authors:** Mary A Lockwood, Ilya Noginskiy, Madhumathi Kalavar

**Affiliations:** 1 Internal Medicine, NewYork-Presbyterian Brooklyn Methodist Hospital, Brooklyn, USA; 2 Oncology, NewYork-Presbyterian Brooklyn Methodist Hospital, Brooklyn, USA

**Keywords:** primary breast cancer, hiv/aids, diffuse large b cell lymphoma, primary breast lymphoma, breast cancer, lymphoma, aids, hiv

## Abstract

Primary breast lymphoma (PBL) is not a commonly seen subtype of breast cancer, and it is also unusual for an extranodal variant of diffuse large B-cell lymphomas (DLBCLs) to appear in the breast. In this case report, we recount the presentation of painful masses in the right axillary and right breast regions in an acquired immunodeficiency syndrome (AIDS) patient, shortly after a mammogram described her breast lesion as BI-RADS 3, probably benign, in the breast imaging reporting and data system. This case demonstrated that painful breast and axillary masses in an acquired immunodeficiency syndrome (AIDS) patient can grow quickly, be misdiagnosed, and require an expedient workup, as extranodal DLBCL can be a debilitating disease.

## Introduction

Primary breast lymphoma (PBL) is a rare type of extranodal lymphoma, causing only 0.4% of breast cancers [1]. A diagnostic criterion for PBL was proposed by Wiseman and Liao: 1) the presenting site of the lymphoma should be the breast; 2) there should be no history of lymphoma or signs of widespread disease at the time of diagnosis; 3) the lymphomatous proliferation should be in close relation with breast tissue histologically; and 4) ipsilateral lymph nodes may be involved and be considered as long as they arise simultaneously with the breast mass [2]. PBL is rarely diagnosed on routine mammogram screenings but, instead, is usually detected once a mass has already been felt [3].

The association between human immunodeficiency virus (HIV) infected individuals and lymphoma has been widely studied. Given this population’s susceptible immune system, they are at higher risk of developing a lymphoma. Since the introduction of highly active antiretroviral therapy (HAART), the incidence in this population has decreased, especially in those with undetectable viral loads [4]. The following is a case of rare PBL occurring in an HIV-positive individual with an undetectable viral load, which was initially perceived as a non-malignant lesion.

## Case presentation

**Figure 1:**
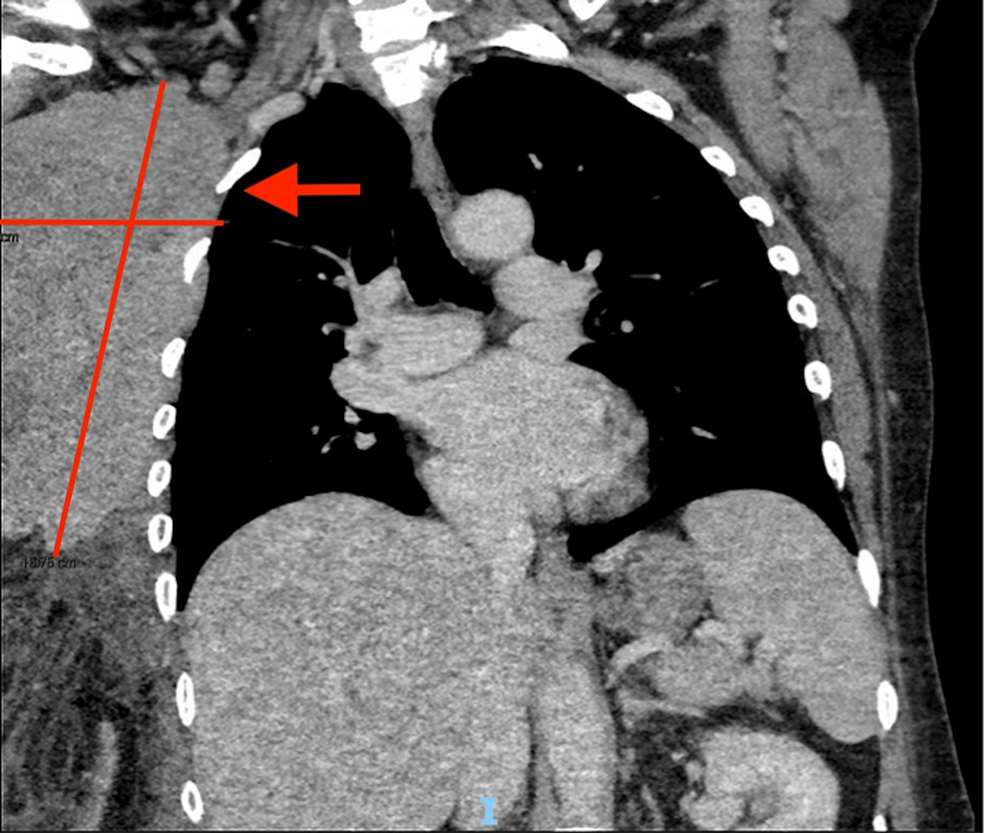
Chest CT scan showed the right breast and an axillary mass of 7x14cm in this plane (arrow)

Our patient is a 62-year-old female with a history of HIV on HAART and a remote history of intravenous drug abuse who presented to the emergency room with a progressively enlarging right axillary mass associated with pain, swelling, and redness. She had concurrent right breast swelling and pain as well. The patient reported regular mammogram screenings and denied any history of malignancy. Additionally, she was compliant with HAART. Approximately two months prior during evaluation for right chest pain, the patient was found to have a 7x6x9cm right chest mass on a CT scan that was relayed as a boil. She was lost to follow-up. She later presented again due to recurrent pain. The patient underwent a mammogram showing diffuse right breast thickening and edema, a solid cystic mass measuring approximately 5.5cm in the right axilla. This finding was given BI-RADS 3, probably benign, in the breast imaging reporting and data system. She presented to the hospital three days following the mammogram for the above complaints. On physical exam, she had a firm and tender mass in the right axillary that was limiting her range of motion. Her right breast had a firm mass in the outer quadrants. At this time, the patient’s CD4 count was 157, and her viral load was undetectable. She underwent a CT scan of the chest, which showed a 10x7x14cm right breast and axillary mass with extension into the pleura (Figure 1).

Staging CT scans did not show any lymphadenopathy or lesions in other organs. A breast and axillary node biopsy was done showing Ebstein-Barr virus (EBV)-positive DLBCL with the germinal center B-cell (GCB) subtype. The patient was initiated on R-EPOCH (rituximab, etoposide, prednisone, vincristine, cyclophosphamide, and doxorubicin) therapy. At this time, due to the patient's concurrent HIV, the decision was also made to treat the patient with prophylactic intrathecal methotrexate inpatient until her lumbar puncture studies resulted. The patient was discharged with a continuation of therapy. The patient has also been referred to radiation oncology for likely initiation of radiotherapy.

## Discussion

Breast cancer is the most frequent malignancy in women worldwide. In most cases, the malignancies arise from epithelial or stromal cells within the breast. Approximately 0.4% of breast cancers are PBLs, in which case the mass arises from the lymphoid tissue [5]. PBLs can be of different histological subtypes, arising from either B or T cells, with the most common being DLBCL, occurring at a rate of 45% to 79% [1,6].

The clinical presentation of PBL is the same as other breast carcinomas, with the most common presentation being a breast lump. Our patient presented with pain and palpable adenopathy, which is only seen in 12% and 25% of patients, respectively [7]. The DLBCL subtype of PBL can be one of the most aggressive subtypes, as it is rapidly enlarging. At presentation, primary breast DLBCL often presents as multiple breast masses, diffuse breast enlargement, and/or enlarged axillary lymph nodes. Generally, imaging in PBL is nonspecific, as seen in our patient [7]. PBL is rarely discovered by screening mammography, as it usually appears as a well-defined mass with benign characteristics, as seen in our patient’s mammogram with BI-RADS 3 [3].

The presence of HIV infection has been associated with an increased risk of many cancers. Patients with HIV have impaired cellular immunity, and therefore have a higher incidence of developing cancer, especially lymphoma [8]. Compared with the general population, HIV patients have a much higher risk of non-Hodgkin's lymphoma (NHL) with a standardized incidence ratio of 11.15 [9]. With progressively decreasing CD4 counts, the risk of NHL goes up in HIV patients [10]. Since the introduction of HAART, the incidence of lymphoma in HIV-positive patients has decreased, especially in those with undetectable viral loads [4].

The presented case demonstrates that, although rare, patients with HIV are at increased risk of PBL, even with an undetectable viral load. Given that PBL imaging findings are nonspecific and the lesions often appear benign on mammograms, proceeding with caution in patients with impaired immune systems is recommended. It is important to be mindful of PBL, especially the DLBCL subtype, as these masses are often more aggressive and present with a comparatively large lesion [5].

The mainstay of treatment for PBL has been chemotherapy, usually CHOP (cyclophosphamide, doxorubicin, vincristine, and prednisone) [7]. Earlier studies focusing on the addition of rituximab have been divergent, with one demonstrating the incidence of CNS relapse in DLBCL decreased and the pattern changed with the addition of rituximab, although it was not completely eliminated [11]. Regarding surgery, a meta-analysis revealed that mastectomy offered no role in survival and can even increase the risk of death [5,12]. Two studies have demonstrated that radiotherapy sequentially with anthracycline-based therapy (without rituximab) improved survival in primary breast DLBCL [11,13]. A retrospective Surveillance, Epidemiology, and End Results (SEER) database analysis demonstrated that patients treated with systemic regimens including rituximab followed by radiation therapy had a significantly greater five-year overall survival (OS) compared to those that did not receive radiotherapy [12]. A retrospective analysis on a limited number of patients demonstrated that treatment with dose-adjusted EPOCH-rituximab, alternating with high-dose methotrexate and cytarabine had a higher five-year OS compared to two other regimens [14]. Presently, no definitive guidelines exist for primary breast DLBCL. Treatment has been patient-specific and supported by the latest evidence in terms of superior and least-harmful treatment.

## Conclusions

HIV patients have an increased risk of malignancy. A suspicious breast mass warrants additional workup, including a mammogram or ultrasound and biopsy. Our case presents a case of HIV-associated lymphoma in the breast despite an undetectable viral load. We recommend observing caution in interpreting mammogram reports in patients with a palpable breast mass and HIV. Physicians must have a high suspicion of malignancy in this population to ensure early detection and treatment with chemotherapy or surgery.
